# Mortality predictors in acute pancreatitis admitted to the ICU

**DOI:** 10.1186/cc10995

**Published:** 2012-03-20

**Authors:** P Vidal-Cortés, P Lameiro-Flores, A Aller-Fernández, M Mourelo-Fariña, R Gómez-López, P Fernández-Ugidos, M Alves-Pérez, E Rodríguez-García

**Affiliations:** 1 CHU Ourense, Spain; 2 CHU A Coruña, Spain

## Introduction

Patients diagnosed with acute pancreatitis (AP) are usually admitted to our units. Despite using a lot of scores, none has proved an acceptable yield to identify patients with higher mortality risk. Our purpose is to identify mortality predictors of patients admitted to our ICU diagnosed with AP.

## Methods

We performed a retrospective study in which we analyzed patients diagnosed with AP admitted to a 24-bed ICU between January 2000 and December 2009. Postcardiopulmonary bypass pancreatitis and readmissions were excluded. Demographic characteristics, co-morbidities and parameters included in severity scores (APACHE II, SAPS II, SOFA) were studied. A Cox proportional hazard regression model was used to assess the effect of each variable on patient survival.

## Results

A total of 122 patients diagnosed with AP were admitted to our ICU between January 2000 and December 2009 (68.9% men, mean age: 60.5 ± 14 years); 43.4% were smokers and 41.8% alcohol consumers. The most frequent comorbidity was hypertension (41.8%), followed by dyslipemia (24.6%), cardiac disease (17.2%), DM and pulmonary pathology (13.1%). Solid or hematologic malignancy (10.6%), chronic renal failure (9%) and hepatic pathology (5.7%) were other comorbidities. Biliary etiology was the most frequent (48.5%), followed by alcoholic AP (20.5%) and unknown etiology (17.2%); 3.3% were post-biliary manipulation (surgery or ERCP) AP. The mean APACHE II score at admission was 16.42 ± 7.64. In total, 56.6% patients needed mechanical ventilation, 50.8% vasopressors and 40.2% renal support during their ICU stay. The ICU length of stay (LOS) was 16.55 ± 21.6, hospital LOS 45.39 ± 45.42 days. A total of 28.7% patients died in the ICU, and 38.5% during their hospital stay. We did not find any relation between comorbidities or AP etiology and outcome. Mortality predictors in AP patients were: PaFi relation (-0.007, *P *= 0.006), mean and systolic arterial pressure (-0.39, *P *= 0.019 and -0.038, *P *= 0.001 respectively), pH (-5.641, *P *= 0.001), HCO_3 _(-0.081, *P *= 0.050), creatinine (0.347, *P *< 0.001), urea (0.008, *P *= 0.002), 24-hour diuresis (-0.001, *P *= 0.002) and Glasgow Coma Scale (-0.312, *P *= 0.050).

## Conclusion

Comorbidities and AP etiology are not predictors of ICU mortality. Of the variables included in severity scores, only those related to organ dysfunction (hemodynamic - SAP, MAP, pH, HCO_3_^-^; respiratory -PaFi relation; and renal - Cr, urea and 24-hour diuresis) are ICU mortality predictors in AP patients.

